# Designing Audience-Centered Interactive Voice Response Messages to Promote Cancer Screenings Among Low-Income Latinas

**DOI:** 10.5888/pcd11.130213

**Published:** 2014-03-13

**Authors:** Mary L. Greaney, Maria De Jesus, Kim M. Sprunck-Harrild, Trinidad Tellez, Roshan Bastani, Tracy A. Battaglia, James S. Michaelson, Karen M. Emmons

**Affiliations:** Author Affiliations: Maria De Jesus, Center on Health, Risk, and Society and School of International Service, American University, Washington, DC; Kim M. Sprunck-Harrild, Center for Community-Based Research, Dana-Farber Cancer Institute, Boston, Massachusetts; Trinidad Tellez, New Hampshire Department of Health and Human Services, Concord, New Hampshire; Roshan Bastani, University of California Los Angeles School of Public Health, Los Angeles, California; Tracy A. Battaglia, Boston Medical Center and Boston University School of Medicine, Boston, Massachusetts; James S. Michaelson, Massachusetts General Hospital and Harvard Medical School, Boston, Massachusetts; Karen M. Emmons, Dana-Farber Cancer Institute and Harvard School of Public Health, Boston, Massachusetts.

## Abstract

**Introduction:**

Cancer screening rates among Latinas are suboptimal. The objective of this study was to explore how Latinas perceive cancer screening and the use and design of interactive voice response (IVR) messages to prompt scheduling of 1 or more needed screenings.

**Methods:**

Seven focus groups were conducted with Latina community health center patients (n = 40) in need of 1 or more cancer screenings: 5 groups were of women in need of 1 cancer screening (breast, cervical, or colorectal), and 2 groups were of women in need of multiple screenings. A bilingual researcher conducted all focus groups in Spanish using a semistructured guide. Focus groups were recorded, transcribed, and translated into English for analysis. Emergent themes were identified by using thematic content analysis.

**Results:**

Participants were familiar with cancer screening and viewed it positively, although barriers to screening were identified (unaware overdue for screening, lack of physician referral, lack of insurance or insufficient insurance coverage, embarrassment or fear of screening procedures, fear of screening outcomes). Women needing multiple screenings voiced more concern about screening procedures, whereas women in need of a single screening expressed greater worry about the screening outcome. Participants were receptive to receiving IVR messages and believed that culturally appropriate messages that specified needed screenings while emphasizing the benefit of preventive screening would motivate them to schedule needed screenings.

**Conclusion:**

Participants’ receptiveness to IVR messages suggests that these messages may be an acceptable strategy to promote cancer screening among underserved Latina patients. Additional research is needed to determine the effectiveness of IVR messages in promoting completion of cancer screening.

## Introduction

Cancer screening rates among Latinas are suboptimal. Compared with whites, Latinas have lower screening rates for breast (mammogram within past year, 46.5% vs 51.5%), cervical (74.7% vs79.1%), and colorectal cancer (CRC) (51.2% vs 60.2%) (1). Improving screening rates is important for reducing racial/ethnic disparities in rates of cancer. However, screening can be burdensome, particularly for women who are age-eligible for multiple screenings. Adding to this burden is that different screenings (ie, breast, cervical, CRC) have different recommended screening intervals and that recommendations vary among organizations and have changed over time (2–5). These variations may cause confusion about when screening should commence or how often they should take place (6–8), and, coupled with barriers to screening, may contribute to disparities in screening rates.

Health care organizations often remind patients of overdue screenings via letters or telephone. Such reminders can increase screening rates (9,10), but a reminder system may be difficult to sustain in community health centers (CHCs) that provide care to low-income populations and have limited resources. Interactive voice response (IVR) messages — automated telephone calls that allow recipients to interact with a prerecorded message through speaking or pushing telephone keys — are a promising low-cost strategy for patient reminders. The technology allows participants to hear an initial message and then additional menu options as desired. The decision to access menu options is driven by the recipient, and staff involvement is minimal once the IVR programming is developed. IVR messages can be tailored by language, name, and other variables. Messages are easily updated, can be delivered at designated times, and are inexpensive to deliver. Importantly, IVR messages are viewed favorably in a medical context (11). Although IVR messages have had mixed success in promoting cancer screenings (12–15), success with this technology could increase if more information about how to design IVR messages to meet recipients’ needs was available (16). Thus, we conducted a series of focus groups with Latinas in need of cancer screening(s) to 1) explore perceptions of and barriers to cancer screening and 2) understand audience communication norms related to the design of IVR messages. 

## Methods

We conducted 7 focus groups in Spanish with Latina patients in need of 1 or more cancer screenings. Focus groups were held in August and September 2008. Five groups were conducted with women needing 1 screening (n = 28) and 2 groups with women needing multiple screenings (n = 12). This design allowed us to examine differences in emergent themes between participants in need of 1 or multiple screenings. All participants were patients of a CHC in a city in northeast Massachusetts that is predominately Latino (>75%) (17) and has high rates of poverty and limited resources (18, 19). Approximately 80% of Latinos living in the city receive care at the CHC; most of the CHC patients are Hispanic (90%) and are of Caribbean origin (ie, Dominican Republic, Puerto Rico) (18).

### Recruitment

Eligibility criteria included being female, aged 21 years or older, an active patient of the CHC (≥1 health care visits in previous 24 months), and in need of 1 or more cancer screenings. Age and screening status were determined through the patient’s electronic medical record (EMR). Guidelines from the US Preventive Services Task Force and the American Cancer Society (20–23) were used to determine screening status: 1) women aged 21 to 70 who had not had a Papanicolaou (Pap) test in the previous 27 months (2 years plus a 3-month data-collection buffer) were deemed in need of cervical cancer screening; 2) women aged 42 or older who had not had a mammogram in the previous 15 months (1 year plus a 3-month buffer) were deemed in need of breast cancer screening; and 3) women aged 51 or older who had not had a colonoscopy in the previous 10 years or a fecal occult blood test within the previous 15 months (1 year plus 3-month buffer) were considered in need of CRC screening. Using these criteria, CHC staff generated a list of eligible patients. A bilingual research assistant telephoned eligible patients to explain the study’s purpose, provide study information, determine interest, and confirm ability to participate in a focus group conducted in Spanish.

### Focus group procedures

Focus groups were conducted at community sites (eg, YMCA) convenient to participants; participants provided informed consent and completed a short demographic survey before the focus groups began. A bicultural, bilingual moderator trained in qualitative research methods conducted the focus groups in Spanish using a semistructured interview guide (Appendix) informed by the sociocontextual model (24). The model recognizes the role of social contextual factors in influencing health-related behaviors, such as participating in cancer screening, across multiple levels of influence (eg, individual, interpersonal, organizational). For this study, the guide focused primarily on the individual level. During the focus group, participants listened to prerecorded samples of IVR messages. The guide explored awareness and perceptions of cancer screening, barriers to screening, and reactions to the IVR messages. Discussions were audiorecorded, and each group lasted approximately 75 minutes. Participants received a $40 incentive and a meal; transportation and childcare were available. This study was approved by the institutional review board of the Harvard School of Public Health.

### Analysis

Audio files were transcribed in Spanish by a native speaker; the Spanish transcripts were then translated into English. English transcripts were coded and analyzed using thematic content analysis; we used NVivo software (QSR International Pty Ltd, Doncaster, Victoria, Australia) for analysis. Initial codes were categorized into higher-order codes that reflected emergent themes (25). We also examined whether there were differences in emergent themes in the focus groups with participants needing a single screening and those with participants needing multiple screenings. 

## Results

All study participants (n = 40) self-identified as Latina, mean age was 51.9 (range, 22–86), and 12 (30.0%) needed multiple screens (Table 1). Nineteen (47.5%) participants had less than a high school education. Thirty-six participants (90.0%) reported having a physician or nurse practitioner at the CHC whom they viewed as their regular health care provider. Although all participants reported the ability to converse in Spanish, 7 (17.5%) participants preferred to speak in English.

**Table 1:** Demographic Characteristics of Participants (n = 40) in Focus Groups on Cancer Screening, Massachusetts, August–September 2008

Characteristic	Value^a^
**Age, mean (range), y**	
Entire sample	51.9 (22–86)
Participants in need of a single screening	49.7 (22–86)
Participants in need of a multiple screenings	56.8 (45–65)
**Latina**	40 (100)
**Education**	
Less than high school diploma	19 (48.0)
Completed high school/vocational school	11 (28.0)
Some college or more	10 (25.0)
**Place of birth **	
United States	6 (15.0)
Outside of United States	28 (70.0)
Missing data	6 (15.0)
**Preferred spoken language**	
Spanish	32 (80.0)
English	7 (17.5)
Missing data	1 (2.5)
**Have cell phone or voice mail**	36 (90.0)
**Screening needs**	
In need of single screen	28 (70.0)
In need of multiple screens	12 (30.0)
**Relationship with health care provider**	
**Have a physician or nurse practitioner at community health center who is perceived as a regular health care provider**	36 (90.0)
**How well the regular physician or nurse practitioner knows patient as a person and understands her values and beliefs**	
Not at all	3 (7.5)
A little	9 (22.5)
Somewhat	3 (7.5)
Very well	25 (62.5)

a All values are shown as number (percentage) unless otherwise indicated. Because of rounding, percentages may not total 100%.

### Perceived importance of cancer screening

All participants were aware of cancer screening; most viewed screening as beneficial and understood the importance of early detection. As one woman said, “It is necessary to get a checkup. Even if one feels fine it is still necessary. One is human and can get sick at any time. Prevention is better than regret.” Participants who had been screened spoke of their family, not necessarily a personal desire for health, as their motivation for screening; they wanted to see their children and grandchildren grow up. Some women who reported having a mammogram had been screened only because of the presence of symptoms.

### Barriers to screening

Five primary barriers to screening emerged (Table 2).

**Table 2:** Identified Barriers to Cancer Screening and Representative Quote, Focus Groups on Cancer Screening, Massachusetts, August–September 2008

Identified barrier	Representative quote
Unaware overdue for screening	“I do it [mammogram] I think that one cannot, this is my personal opinion, one cannot stop from doing it, to do for example any type of exam because it hurts.”

Lack of physician referral	“Nothing impedes me [from getting screened for CRC]. It’s just that the doctor hasn’t sent me to have it done.”

Embarrassment or fear of screening procedures	“I don’t go to the [screening] appointment. Or if I do go, once I’m going in like my heart starts racing and then I have to, obviously get undressed and that is when I start sweating. I just get nervous.”
	*“*When I had a female doctor I would go every year but with him [male doctor] I get a little embarrassed. That is what I have, a bit of shame.”
	“The squeeze that they give me. . . . There is no one that can make me do it. My doctor is Hispanic, she is Puerto Rican, and she tells me that it does not hurt and I tell her to forget about it.

Lack of health insurance/insufficient insurance coverage	“I do not have medical insurance through my agency work. And I did not keep up with my mammograms. I did not do it for 2 years. And eventually I received medical insurance, not too long ago, from [Massachusetts subsidized health insurance program], the one that everyone has to have, and when I got that insurance I took advantage of it and made an appointment. “
	“I didn't have any type of medical insurance, and I would have had to pay a lot of money [for mammogram].”

Fear of screening outcomes	“You can go in there thinking nothing is wrong and come out with your whole life being changed.”

**Unaware overdue for screening. **Some participants believed they were current with screenings, although their EMR indicated otherwise. Women were more likely to incorrectly think they were current with cervical cancer screening than with breast cancer or CRC screening.

**Lack of physician referral. **One reason cited by patients for not being current was lack of notification by their physician that they were due for screening or the physician not having ordered the screening. Some women in need of CRC screening stated their physician had not initiated a colonoscopy referral, an essential first step of the colonoscopy process. Not all women who knew the age recommendations for screening wanted to ask for a referral.

**Embarrassment or fear of screening procedures. **This theme emerged in all groups but appeared to be of greater concern to women needing multiple screenings. Participants spoke of being embarrassed to participate in screening and said this delayed or prevented scheduling or attending appointments. Several participants indicated they become embarrassed if the physician conducting the cervical examination is male, which translated into reluctance to get screened. A few noted they would not want to have a male physician perform a colonoscopy. Additionally, participants were fearful about pain associated with mammograms and Pap tests because of personal experiences or what they had heard from others.

**Lack of health insurance or insufficient insurance coverage. **Women who had no insurance, regardless of the number of needed screenings, spoke of a lack of health insurance as delaying or preventing screenings. These women felt they would take action to get screened if they had adequate insurance.

**Fear of screening outcomes.** Although this theme emerged in all groups, women in need of a single screening appeared to be more fearful than woman in need of multiple screenings that their examination would reveal cancer. This fear delayed or prevented screening for some women, especially those who had a family history of cancer. For a subset of women needing multiple screenings, preventive tests were less pertinent in their lives because of their belief in a predetermined fate. Some felt that that it was in God’s hands whether they got cancer. And, if they were diagnosed with cancer, God would determine survival. Still, most of these women viewed screening favorably and planned to get screened.

Other barriers mentioned by a few participants included a lack of transportation or daycare and forgetfulness. Some woman felt they were not at risk for cervical cancer because they were not sexually active or they had had a hysterectomy.

### IVR messages

**Receptivity to IVR messages.** Whether in need of a single or multiple screenings, most women were receptive to receiving IVR messages about cancer screenings on their cell and home telephone, saying the messages demonstrate that their provider cares about them. A few women wanted CHC staff to continue making reminder calls, because they valued this personal connection. Some preferred mailed reminders; they liked written documentation that they could refer to if needed.

**Delivery of IVR messages.** Among all groups, participants agreed that IVR messages should be in Spanish and that to ensure confidentiality recipients should enter a personal identifier (eg, year of birth) to hear the message. To accommodate people who have difficulty keying in numbers, participants thought that message recipients should also be able to speak the identifier. Women who had caller ID wanted the IVR identifier to be the CHC’s telephone number, because they would answer calls from the CHC but ignore calls from blocked or toll-free numbers. Participants preferred IVR messages to be recorded by a member of the clinic staff (eg, receptionist) who was a native speaker and representative of their community.

Most women were receptive to receiving multiple IVR calls; they felt that multiple calls would encourage them to make a screening appointment. Several participants felt that if they were to receive multiple IVR reminder calls that the first call should provide detailed information and subsequent calls should be brief.

**Message content.** Participants preferred brief messages but felt that messages should name the needed screening(s), emphasize the importance of screening, and explain why screening is important for Latinas. Several participants thought that messages should include options to hear additional information about 1) details about the needed screening (eg, preparing for screening); 2) clinic hours (address and telephone number were deemed unnecessary); and 3) transportation options. Participants felt IVR messages needed to be culturally tailored and linguistically appropriate. As 1 participant said,

For example, they say, ‘If your response is yes, then press the star button.’ ‘Star’ is what Americans say but on the telephone there is no star; it is an asterisk. It all depends on who you are speaking with. If the person you are speaking with understands that that is a star then let’s press the star, but if the person understands it’s an asterisk then he/she will begin to look for a star.

Another woman explained, “There are many older people that don’t know what the number sign is.”

Among women needing multiple screenings, participants were equally divided as to whether they preferred separate messages for each screening or 1 message for all screenings. A few women felt that receiving an IVR message about all needed screenings may cause worry. Most participants, regardless of number of needed screenings, felt that messages left on voicemail should not name the needed screening(s), because messages that name screenings may cause family members undue concern.

Most participants believed that receiving IVR messages would prompt them to schedule an appointment; some women said they would feel guilty if they did not make an appointment. On the other hand, a few women said that IVR messages would not motivate them to schedule an appointment, because they did not intend to get screened.

## Discussion

We successfully recruited Latinas in need of cancer screening into our qualitative study and found that most participants in our focus groups were receptive to the idea of receiving multiple IVR messages to prompt scheduling of needed screenings. Our findings indicate that messages tailored to a Latina audience would have more value than generic messages and that messages identifying the screenings needed and addressing certain concerns may be important to encourage Latinas to seek screening.

Other research shows that Latino patients will access health education information via telephone (26). Thus, IVR messages are likely to be an acceptable strategy to promote the scheduling of needed screenings and initiate the screening process. Although the barriers identified in our study are generally in line with barriers reported elsewhere (27,28), it was important to obtain this information from our target population to inform the design and tailoring of the IVR messages.

Our findings can guide the development of IVR messages to promote cancer screening among Latinas. First, IVR messages should be delivered by a native speaker from the region represented by the target population. Using a locally recognized voice (eg, person who records CHC’s telephone recordings) and a customary greeting would likely increase the level of call acceptability and meaningfulness. Second, the telephone number that appears in caller ID should identify the CHC; this may reduce the number of hang-ups. Third, messages should specify the date of the patient’s last screening and clearly state that her physician, EMR, or other CHC records indicate screening is overdue. Many study participants incorrectly believed they were current with needed screenings. Because screening recommendations change and such changes create confusion about when to get tested (6,7), CHC’s practice guidelines should also be stated. Fourth, messages should 1) emphasize the preventive role of screening (to alleviate concerns that the call is relaying information about a health problem), 2) recognize and address the fear and embarrassment often associated with screenings, and 3) include the idea of family as a motivator for screening. For some participants in our study, a lack of insurance or inadequate insurance prevented screening; screening rates among women who have insurance are higher than among women who do not (29,30). Thus, IVR messages should include an option that allows recipients to connect with CHC staff to discuss insurance-related questions.

Tailoring IVR messages and identifying all screenings needed in 1 message may be better strategies than creating generic messages and identifying each screening needed in separate messages, especially because the additional costs of following these strategies would be nominal. Fear of screening procedures was a barrier identified by all focus groups but was especially relevant for women needing multiple screenings. Messages could be crafted to let recipients know that although multiple screenings are needed, they can be completed at different times.

Focus group participants, especially those needing a single screening, expressed fear of screening outcomes, and this fear prevented or delayed screening. This finding reinforces the importance of IVR messages that focus on the long-term, beneficial outcomes (ie, early detection may lead to better prognosis) of completing screenings. Our study also highlights the importance of addressing provider-level factors in IVR messages (eg, setting up a referral system for needed screenings) and encouraging patients to take action to get screened. Some participants were waiting for their provider to recommend screening; IVR messages should encourage patients to discuss screening with their providers. IVR messages alone are likely to have a limited effect on cancer screening rates among CHC patients unless they are implemented with other systems-level efforts. Educational campaigns such as bilingual flyers posted in the examination room are easy to implement and may be an effective way to reach patients. In addition to simple efforts, more comprehensive (and likely more effective) strategies should be considered: for example, clinic-supported systems-level interventions such as tailored EMR templates and provider education.

This study has several limitations. Because our study was conducted to inform the development of an intervention to be implemented at 1 CHC, we recruited participants only from this site. Although the CHC serves the entire city, it serves a primarily Latino population of Caribbean origin, thus limiting the generalizability of the study results. Although all participants were able to converse in Spanish, 7 participants preferred to speak English; those who preferred English may be more acculturated than those who preferred Spanish. We did not assess level of acculturation or explore how it may affect personal views about cancer screenings. Women who participated in our study may have been different from women in need of cancer screening(s) who elected not to participate. Finally, we could not access EMRs to determine whether participants who reported that they had not received a provider referral (needed to begin the colonoscopy process) were accurate in their recall.

Given that prompting methods such as letters and telephone calls made by staff are often difficult to sustain, IVR messages hold promise as a feasible, cost-effective strategy to promote cancer screening among underserved Latina patients. As CHCs continue to develop information technology systems, opportunities exist to increase the systematization and efficiency of e-health (eg, IVR messages, text message) strategies to remind patients of overdue cancer screenings that are both sustainable and scalable. These efforts can reduce organizational burden cost-effectively, especially if the EMR can automatically initiate IVR calls according to individual need. Reducing the amount of staff time needed to confirm screening needs and initiate call reminders would free up time to focus outreach efforts on patients who do not respond to automated messages. Future research is needed to test the effectiveness of IVR messages in promoting the scheduling and completing of cancer screenings.

## Appendix. Sample Focus Group Questions

[Moderator = M] What comes to your mind when you hear the term “cancer screening”?

[M] What are your thoughts about getting tested for (type of) cancer?

[M] Have you ever heard of a (cancer screening test)? If so, what do you think of this test?

Probes: What things keep you from getting a mammogram? What are your fears? Please explain. What are your concerns? Please explain.

[M] We are going to change directions slightly now to talk about making screening appointments. The CHC is going to be making telephone calls to people who need to have cancer screening tests and ask them to make an appointment. Here’s an example of what that message might sound like:

MESSAGE PART 1: This is the (CHC). Is this (patient’s name)? To ensure your confidentiality so that only you receive this reminder message, please enter the month and day of your birthday in numbers followed by the pound sign. For example, if your birthday is February 14 please press 0214 followed by the pound sign. (Birthday entered)

MESSAGE PART 2: This is a reminder message that you are due for (cancer screening). Please call (CHC) to make an appointment at (phone number) between (clinic hours). If you do not call the CHC to make an appointment for your (cancer screening), we will call again at another time. Thank you. To hear the message again, please press 1 now. To hear hours of operation again, please press 2 now. To you need to find out where the CHC is located, please press 3 now. Thank you and good bye.

[M] What do you think about the message you just heard asking you to make an appointment to get your [specific screening test]?

Probes: What did you like? What did you dislike? What would you change?

[M] Do you think a message like this would get you to make an appointment to get your [specific screening test]? Why or why not?

[M] What other information could be included in this call that would help you decide to make an appointment for the test?

Probes:

Would you specify the type of cancer screening test or would have the message state that you have a “medical appointment”? Why?

Would you want the reminder message to include information about the importance of the screening test for your cultural community? Why or why not?

[M] If a message is left on your voicemail, how do you think others in your home would respond to hearing it?

[M] Who do you think would be the ideal person leaving you the message?

Probe: Would you prefer a male or female to be the person leaving you the reminder message? Why?

[M] The second kind of message you will hear now is for after you have made an appointment — to remind you to go. Here’s an example of what that message might sound like:

PLAYED MESSAGE: Hello. This is the [CHC]. Is this [patient’s name]? This is a reminder for your appointment at [CHC] for your test on [date] at [time]. If you have any questions, feel free to call [CHC] at [phone number] between [clinic hours]. To hear the date and time of your appointment again, please press 1. To hear the hours of operation again, please press 2 now. If you need to find out where the CHC is located, please press 3 now. If you need instructions as how to prepare for, please press 4 now. Thank you and good bye.

[M] What do you think about the message you just heard?

[M] What other information would you include in this message?
